# Integrated Approach to Upper Body Shaping: Long Time Results

**DOI:** 10.1007/s00266-025-04795-y

**Published:** 2025-03-28

**Authors:** Huseyin Emre Ulukaya, Sabri Ozturk, Kamuran Zeynep Sevim, Burak Tunahan Ekincikli

**Affiliations:** Department of Plastic, Reconstructive and Aesthetic Surgery, Sisli Hamidiye Etfal Training and Research Hospital, University of Health Sciences, Istanbul, Turkey

**Keywords:** Autologous augmentation, Massive weight loss, Postbariatric patients, Spiral flap, Upper body lift

## Abstract

**Background:**

As the frequency of bariatric procedures rises, so does the demand for breast contouring operations. Massive weight loss results in ptotic breasts that are challenging to shape. However, concentrating solely on the breasts in the upper body leads to a state of imbalance. In this study, we aimed to add additional volume to the breast tissue by utilizing the lateral chest folds/back folds with combined upper body lifting procedures and show the long-term results.

**Methods:**

Between January 2018 and June 2024, 24 patients presented to our clinic with a complaint of breast deformity due to massive weight loss. They were evaluated as a whole. Upper body lift and autoaugmentation mastopexy with a lateral thoracic region perforators-based flap were planned in a single stage. Dorsal soft tissue was also utilized for autoaugmentation in selected patients. The viability of the extended dorsal flaps was examined with the intraoperative indocyanine green angiography.

**Results:**

The body contouring surgery was performed an average of 2.2 years after bariatric surgery. Autoaugmentation with extended fasciocutaneous flap procedures was performed in combination with other upper body lifting procedures according to the patient’s needs. The postoperative follow-up period was 1 month to 5 years.

**Conclusions:**

In postbariatric patients, focusing only on the breast leads to aesthetic disharmony in the upper body. Using subcutaneous tissue in the autologous augmentation method eliminates sagging in the upper body and provides the desired volume to ptotic breasts. The five-year follow-up results demonstrate that this method provides long-lasting, satisfying results with minimal complications.

**Level of Evidence IV:**

This journal requires that authors assign a level of evidence to each article. For a full description of these Evidence-Based Medicine ratings, please refer to the Table of Contents or the online Instructions to Authors www.springer.com/00266.

**Supplementary Information:**

The online version contains supplementary material available at 10.1007/s00266-025-04795-y.

## Introduction

Morbid obesity is associated with numerous comorbid conditions that strongly affect the quality of life and shorten the lifespan [1]. Several approaches are used to treat morbid obesity, including medication, diet, exercise and bariatric surgery [2]. The epidemic of obesity has been recently followed by a new epidemic of massive weight loss (MWL) [3]. In the study, those who lost more than 50% of their weight were considered MWL patients.

In those patients, body contour deformities involve almost all areas of the body. However, skin quality is particularly affected. Decreased resistance, greater skin elasticity, degradation of the collagen system and thinning of the dermis can all worsen any surgical outcome and increase the rate of complications [4]. The excess tissue makes it difficult to maintain proper hygiene that results in skin infections, intertrigo and dermatitis, which may also occur in the sagging body parts. Furthermore, MWL affects patients’ psychological, social and sexual well-being as well [3].

During our daily practice, an increasing number of women seek breast reshaping after MWL. However, focusing only on the breast in the upper body leads to disharmony. Therefore, body contouring procedures shall be tailored to each patient in staged operations.

A strategy built on implants is commonly used to treat volume loss in breast, but implants do have some shortcomings. After MWL, traditional mastopexy operations have not been able to provide a breast with a long-lasting and aesthetic form. In 1975, Ribeiro first described augmentation mastopexy with autologous tissue [5]. His method uses excess tissue of the lower and center poles of the breast to serve as a natural implant. Graff and Biggs subsequently detailed modifications to the technique [6, 7]. These techniques, however, are not used in patients with MWL because there is not enough breast tissue.

**Fig. 1:**
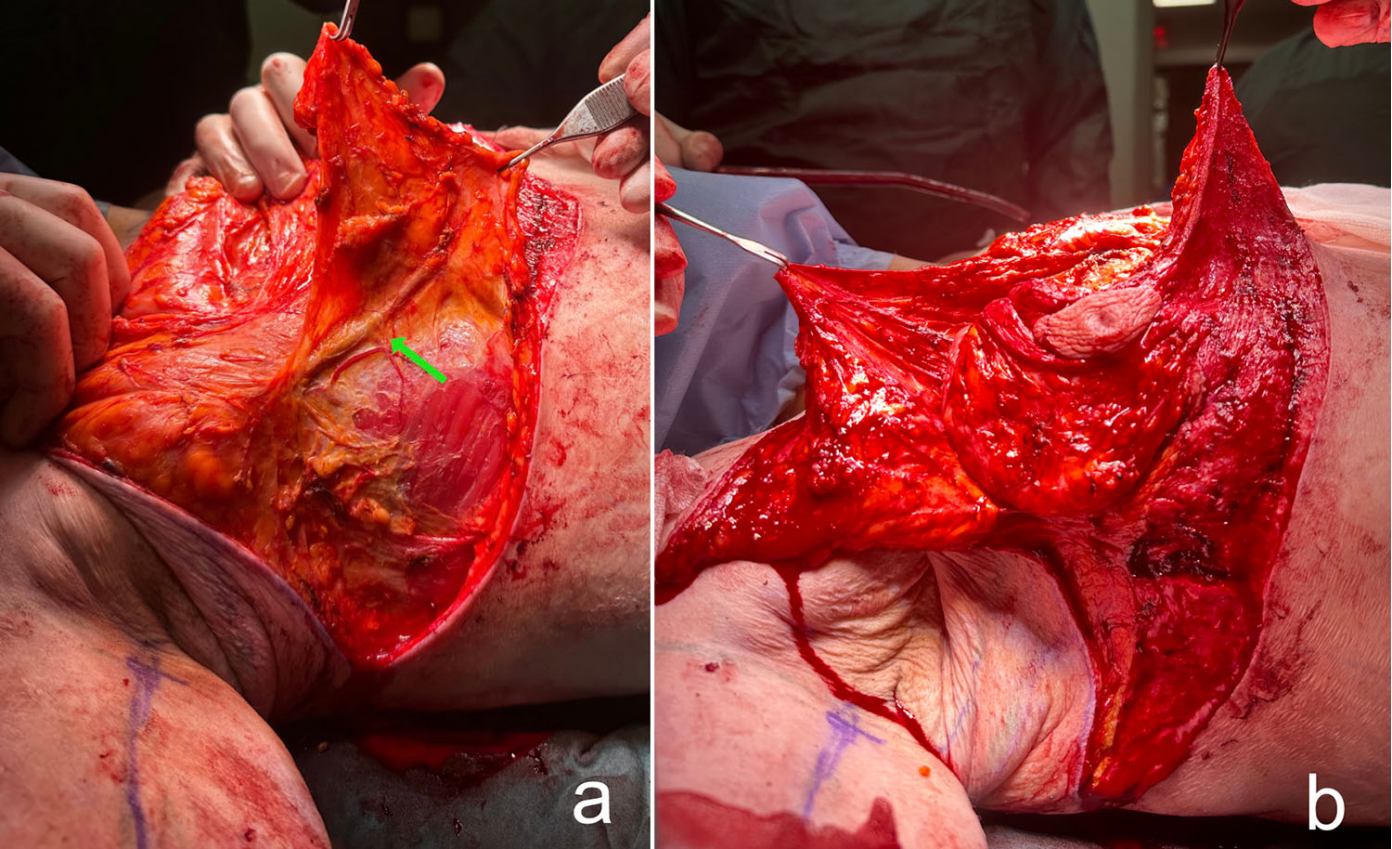
**a** The lateral flap supplied by the lateral thoracic and intercostal perforators is elevated. The large caliber pedicle is indicated by the green arrow. **b** The medial flap, supplied by the anterior intercostal perforators, is elevated over the pectoral muscle

Lateral thoracic perforator flaps based on intercostal [8, 9] or thoracodorsal [10] arteries were initially used in partial mastectomy defect reconstruction. Subsequently, this experience has been applied to lateral thoracic flaps to autoaugment the MWL breast. In the MWL patients, lateral thoracic region perforators retain their large caliber even after associated subcutaneous fat loss following bariatric surgery (Fig. 1a) [11]. Thus, a deepithelialized fasciocutaneous flap extended to the back can be safely harvested. Rubin described the principles of dermal suspension and total parenchymal reshaping for autoaugmentation of the MWL breast. He used lateral chest rolls for the MWL breast’s autoaugmentation [12]. Hurwitz described a similar technique and called the fasciocutaneous flap extended on the lateral side of the breast the ‘Spiral flap.’ He combined the spiral flap with a reverse abdominoplasty for contouring the breast and chest [13]. In both techniques, random fasciocutaneous flaps are harvested. Because the flap is sufficiently long, it can extend to the pillar of medial breast tissue. Therefore, perforator dissection is not required to increase the arc of rotation.

The aim of this study is to show the feasibility of deepithelialized fasciocutaneous flaps harvested from the axillary region that extend to the back for autologous tissue augmentation mastopexies in MWL patients and present the long-term results of these mastopexies with combined upper body lifting procedures.

## Patients and Methods

During the period of January 2018 and June 2024, 24 patients with MWL who presented to the plastic surgery clinic with the complaint of upper body sagging were included in this study. The study excluded patients who have lost less than 50% of their body weight, underwent breast reduction or augmentation with implants and did not come to follow-up.

Demographic information, body measurements, medical history and pictures of the patients were recorded before the operation. Early and late postoperative patient satisfaction, body measurements and control pictures were recorded.

All patients were asked to cease smoking for at least two months before surgery. This study included patients whose weight remained stable for at least six months.

## Preoperative Marking

**Fig. 2:**
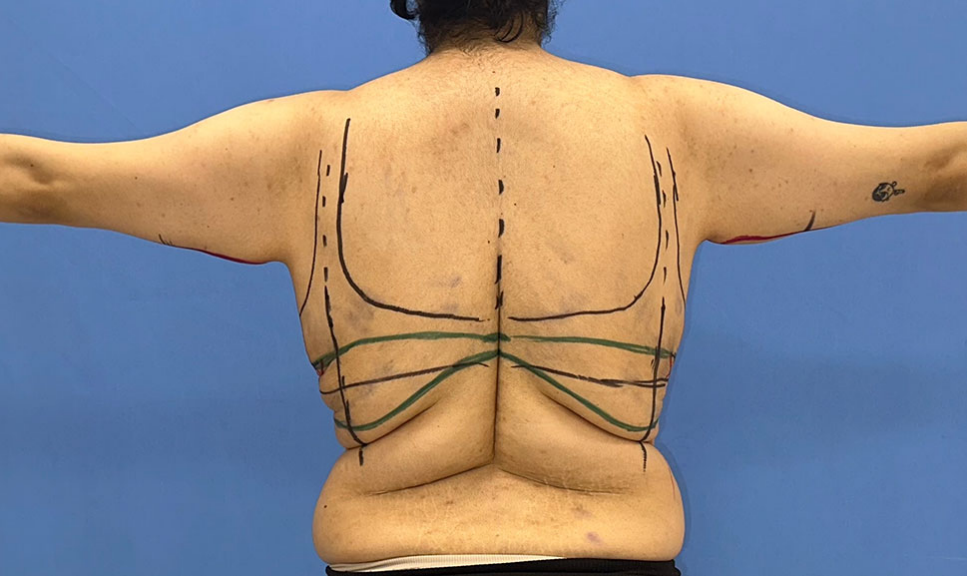
The upper and lower horizontal borders of the patient’s favorite bra were highlighted with a blue marker. The area to be excised is marked with a green marker. The green horizontal line superiorly represents the continuation of the line from the lateral pillar of the breast

**Fig. 3:**
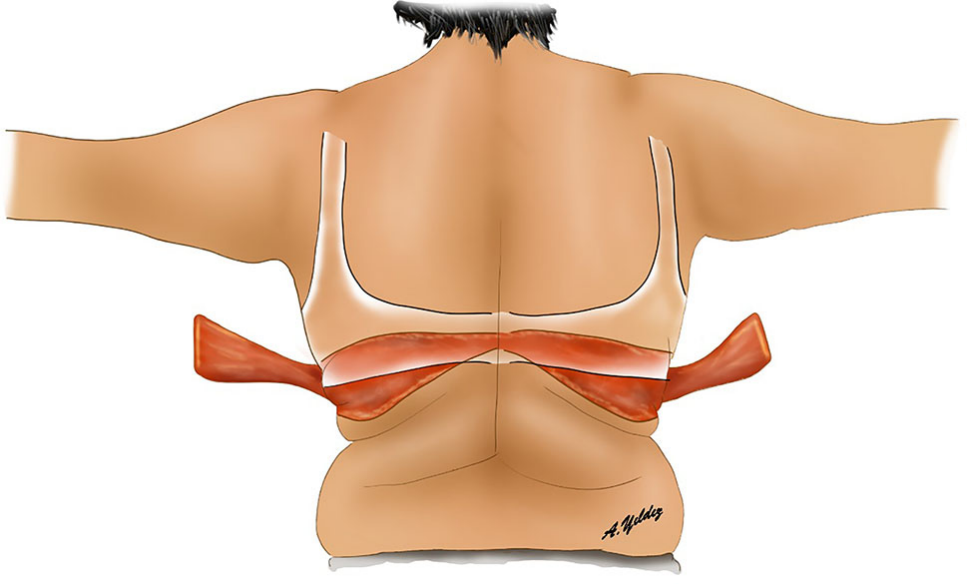
‘Bra-line’ backlift markings and illustration of flaps elevated from the back

**Fig. 4:**
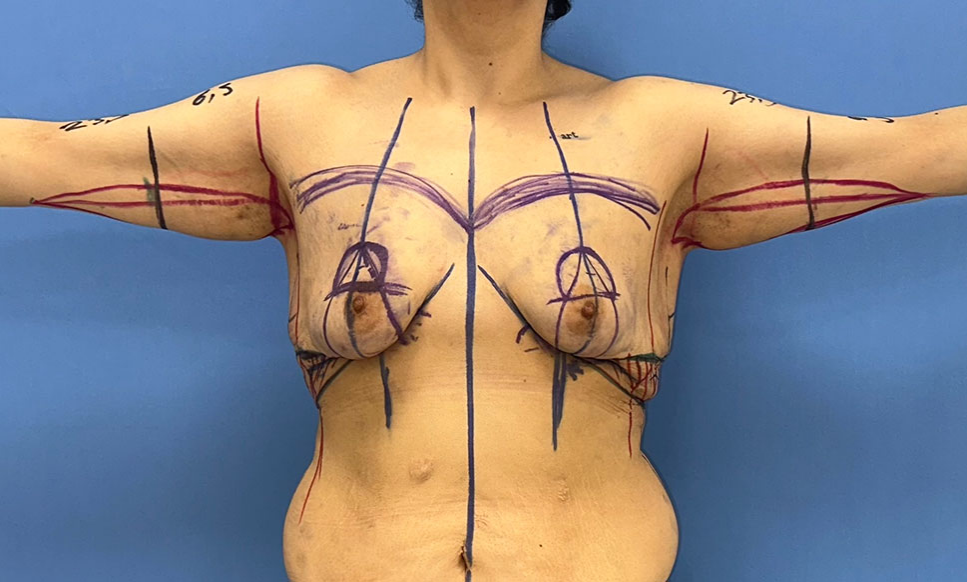
After marking the breast meridian and breast footprint, the new NAC position is decided. Then, a ‘wise’ pattern is drawn

**Fig. 5:**
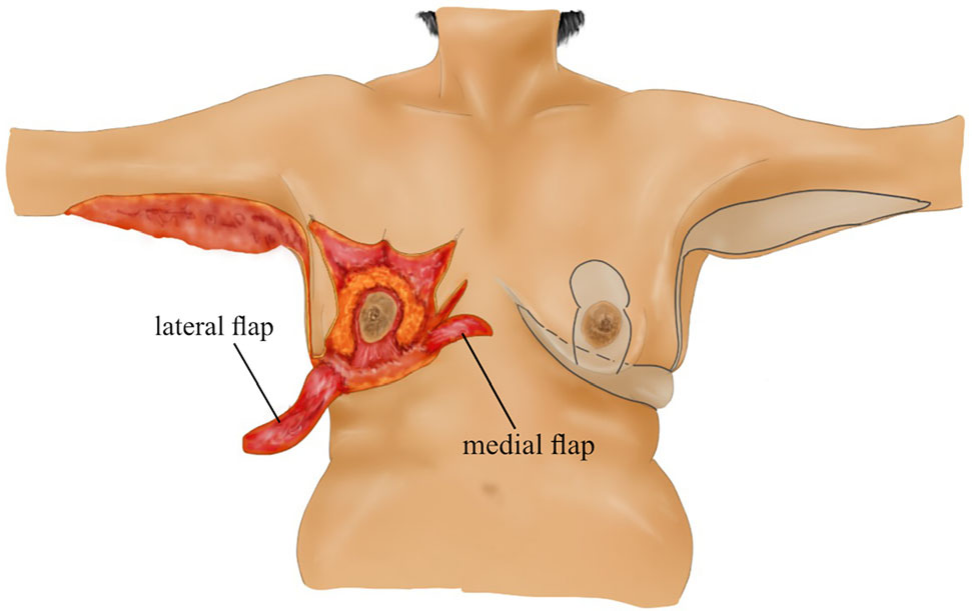
Left breast-planned incision lines. Right breast: elevation of thin skin flaps with preservation of central pedicled breast tissue, illustration of deepithelialized fasciocutaneous medial and lateral flaps

The markings begin with tracing the patient’s commonly worn bra to ensure that the dorsal incision is hidden below the bra. In accordance with the ‘bra-line’ backlift technique, the upper and lower horizontal borders of the bra are marked on the back (Figs. 2,3) [14]. The anterior-, posterior midline, anterior-, posterior axillary line and the breast meridian are drawn. The location of the new nipple-areolar complex (NAC) is then calculated (Figs. 4,5-right breast). The new position of the NAC is set between 18 and 24 cm from the sternal notch to the nipple. After that, a central pedicled wise-pattern mastopexy is planned.

**Fig. 6:**
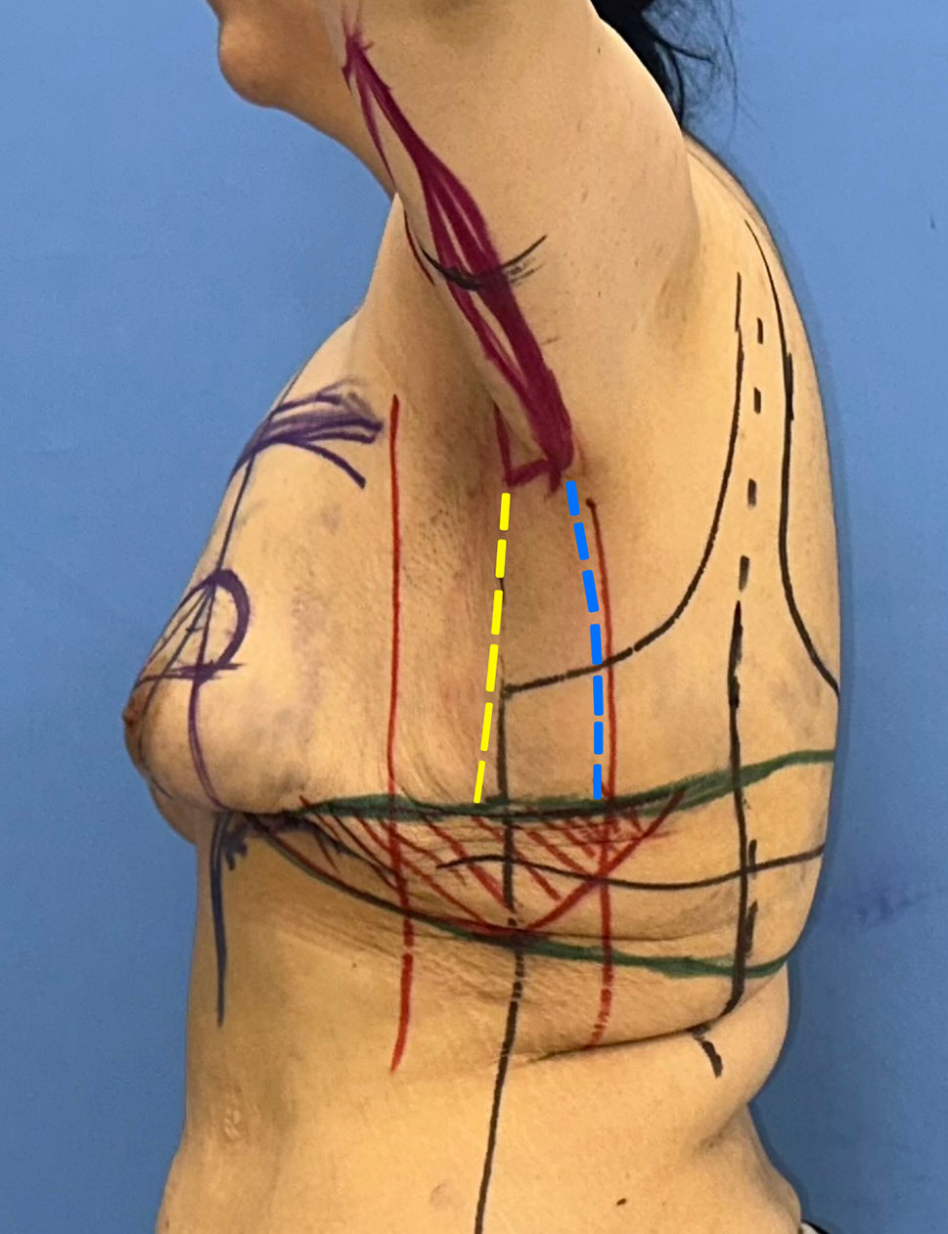
The inferior green marker passing through the inframammary fold is extended laterally. According to the ‘wise’ pattern, the superior green marker from the lateral pillar is extended laterally and dorsally. This green horizontal marker indicates the superior line of excision. The inferior border of the lateral and dorsal folds is determined and marked with a pinching test. The posterior straight line (blue dashed line) passing through the posterior arm is extended inferiorly. It then passes through the posterior axillary fold and is in front of the posterior axillary line. The anterior straight line from the bicipital groove is extended inferiorly through the deltopectoral, axilla and lateral chest. It passes lateral to the lateral pectoral fold (yellow dashed line). The extension of the anterior straight line to the lateral chest is determined by the pinching test. The anterior and posterior straight lines are connected inferiorly with the superior green line

In order to place the deepithelialized lateral chest roll flap, the leg of the lateral pillar is extended posteriorly according to the wise-pattern drawing. If the patient has an upper back roll, the horizontal line passing through the superior border of the lateral chest roll from the lateral pillar is extended to the back to include this back roll (Fig. 6-upper horizontal green line). Bilateral horizontal lines can be connected at the back. The inferior border of the lateral and dorsal folds is determined and marked by the ‘pinch’ test (Fig. 6-lower green line.) For a tension-free closure, the excised tissue should be limited, especially in the dorsal midline area.

Then, for patients with sagging skin of the arms, axillae and lateral chest, the L-brachioplasty technique is utilized (Fig. 5) [15]. The anterior straight line is crossed slightly above the bicipital groove. The line passing through the posterior arm becomes the posterior straight line. By this technique, the scar is positioned on the midposterior arm. The arms are 180° elevated to determine the sagging amount of the skin of the axillae and lateral chest tissue. The anterior straight line from the bicipital groove is extended inferiorly from the deltopectoral through the axilla and lateral chest, passing lateral to the lateral pectoral fold. This line is connected below with the superior line of the bra-line backlift marking (Fig. 6-yellow line). The posterior straight line is determined by the ‘pinch’ test. The line traverses the posterior axillary fold and extends anteriorly to the posterior axillary line. This line is connected below to the superior line of the bra-line backlift drawing (Fig. 6-blue line).

**Fig. 7:**
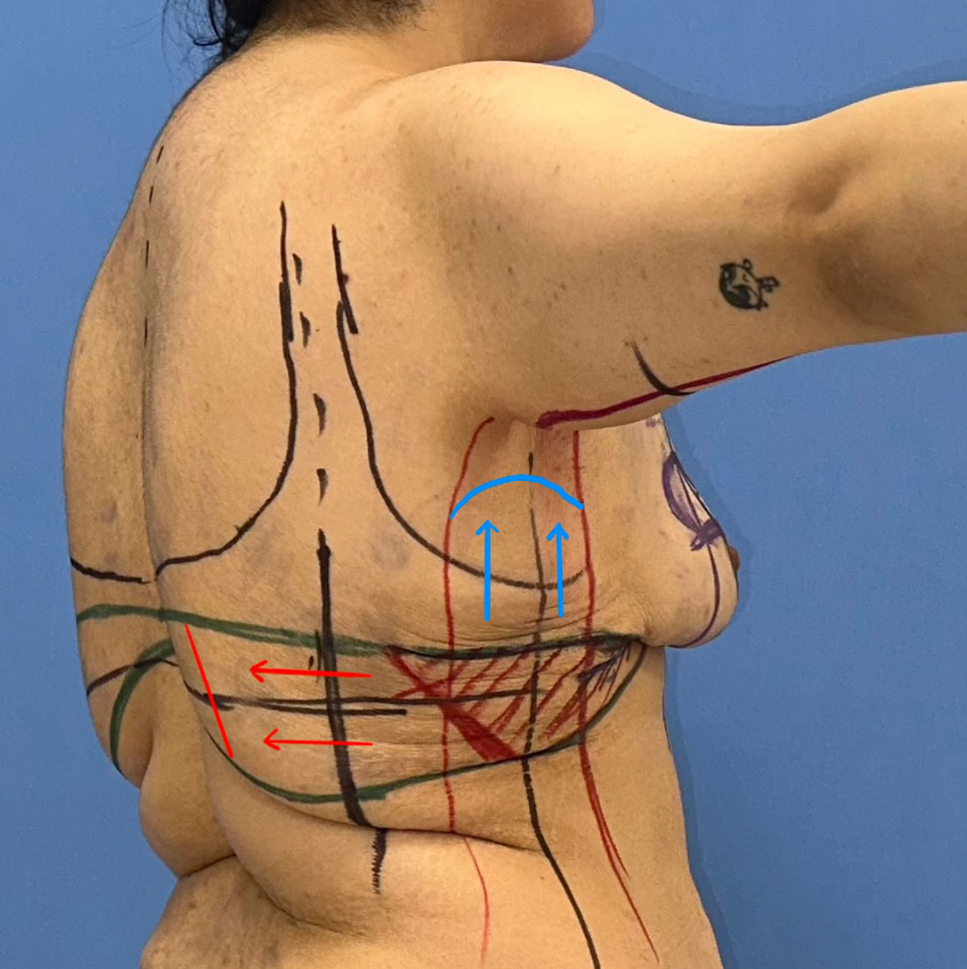
The tissue to be used in autoaugmentation is decided according to the patient’s needs and subcutaneous tissue. This area is crosshatched in red

Finally, the desired breast volume and the patient’s subcutaneous tissue are reviewed. If needed, a deepithelialized chest roll flap of the breast can be harvested either from the lateral chest folds or extended dorsal folds. The flap to be used for autoaugmentation is crosshatched in Fig. 7.

## Surgical Technique

**Fig. 8:**
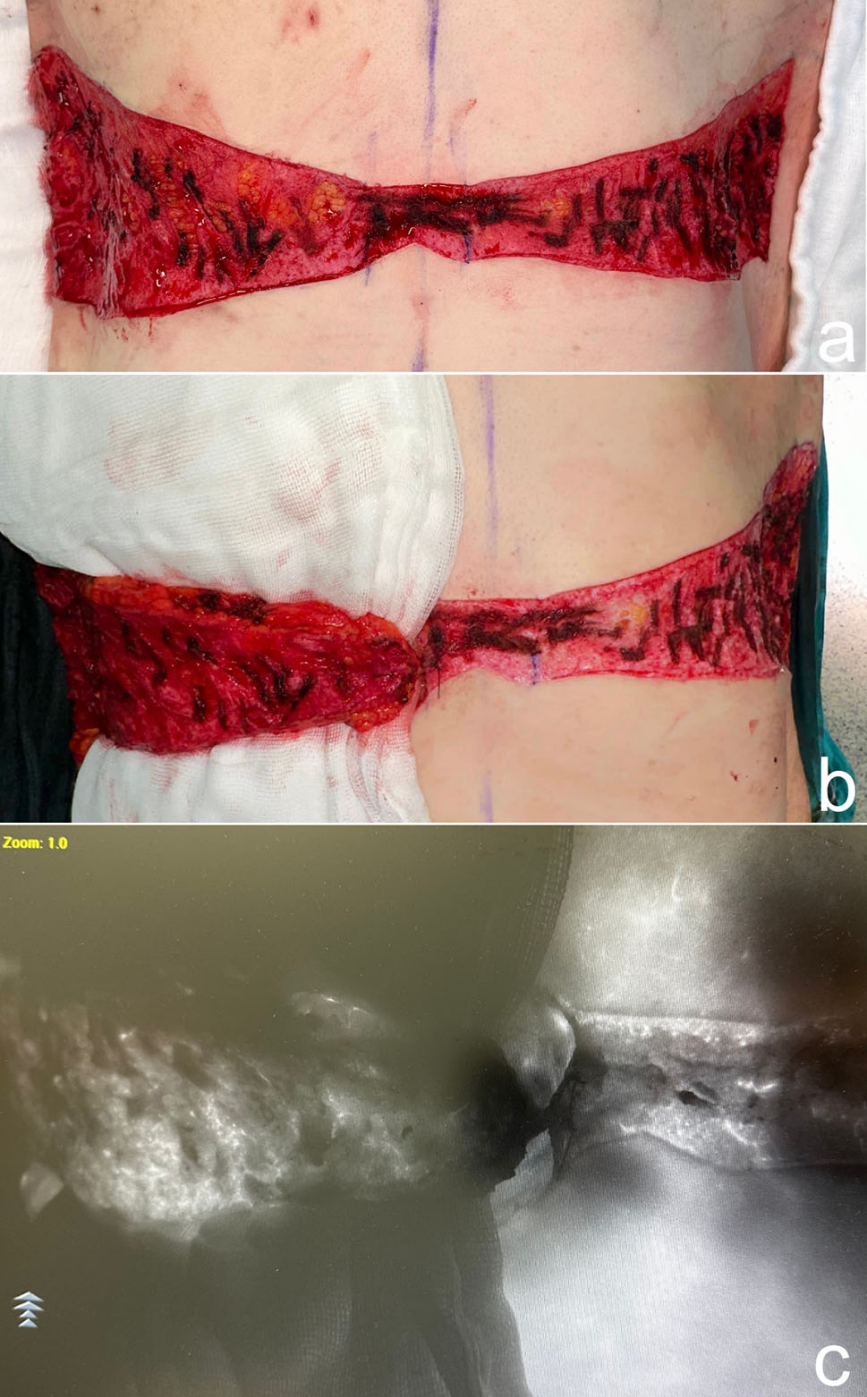
**a** The deepithelialized extended flap on the back. **b** The flap is elevated and a compress is passed underneath. **c** Laser-assisted indocyanine green angiography imaging demonstrates perfusion in the elevated flap with brighter areas. Perfusion up to the tip of the elevated flap, which corresponds to the dorsal midline, is seen

The surgery starts in prone position under general anesthesia. Thus, the dorsal and lateral chest portions of the flaps to be used for autoaugmentation can be harvested. Local anesthetic is injected into the incision lines. Then, the area planned to be used for autoaugmentation is deepithelialized. The extended deepithelialized flaps are elevated over the latissimus dorsi muscle fascia and then over the serratus anterior muscle until the anterior axillary line is reached (Fig. 3). Perforators of the flap are confirmed using a handheld Doppler and/or a laser-assisted indocyanine green angiography (Fig. 8). The viability of the tip of the flap is checked. The remaining dorsal and lateral chest tissue is resected over the superficial fascia. The skin is closed in a double-layer manner with absorbable sutures. The extended fasciocutaneous flaps are wrapped with wet gauze and draped accordingly.

**Fig. 9:**
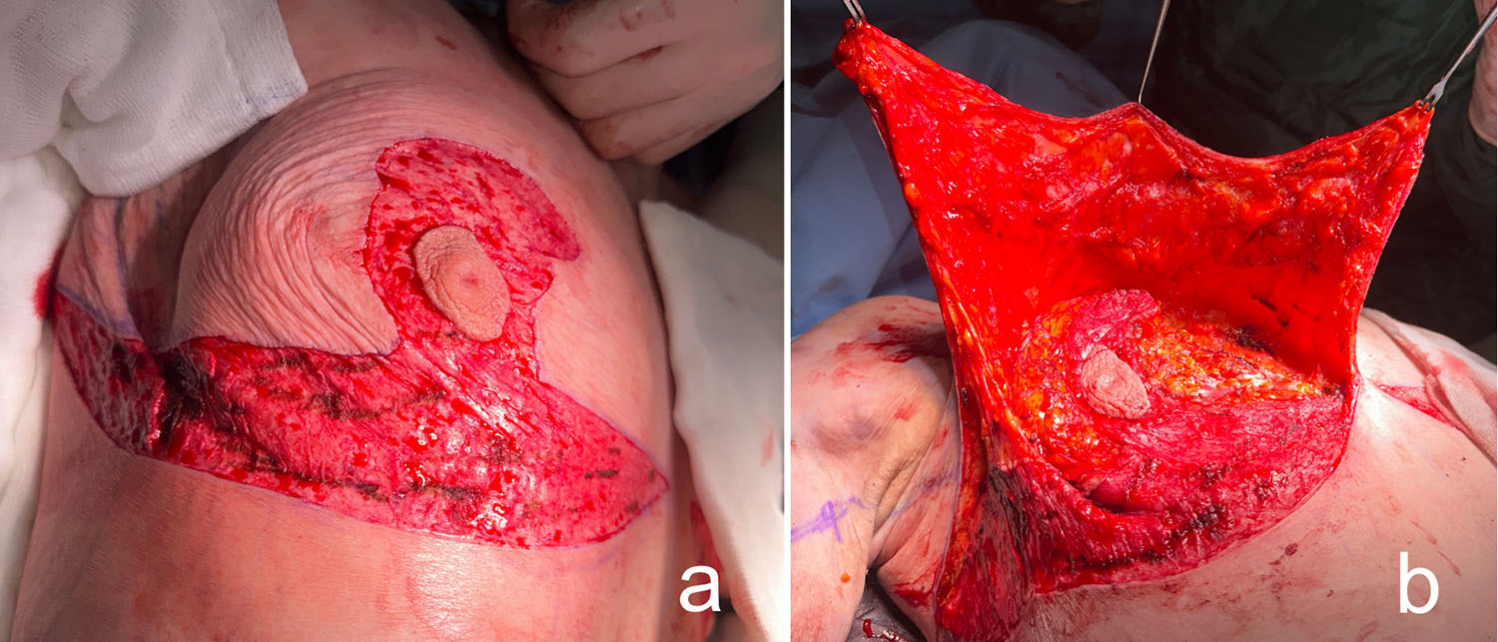
**a** The area outside the NAC in the wise pattern is deepithelialized. **b** Thin skin flaps are elevated, preserving the central and inferior pedicled breast tissue

**Fig. 10:**
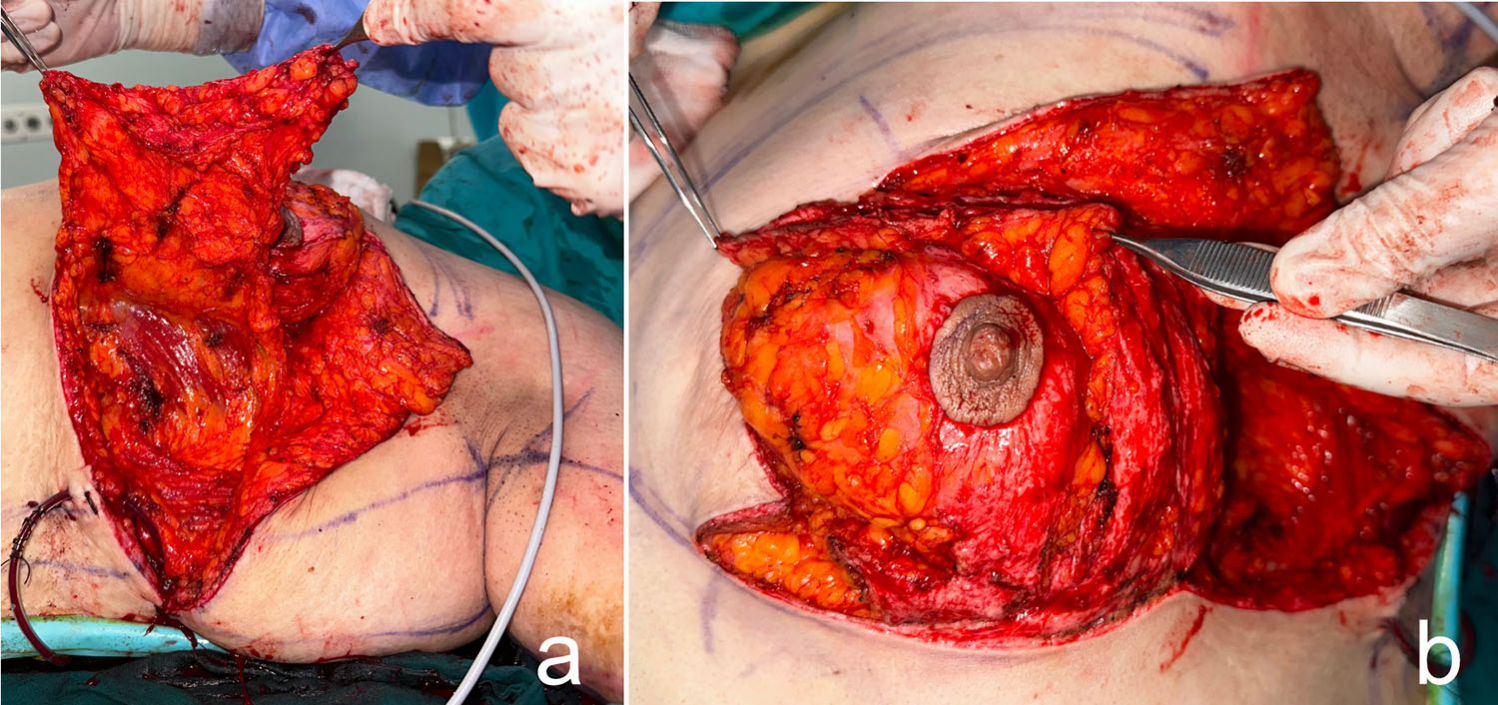
The bulky flap shown in (**a**) is folded over the central breast tissue as in (**b**) and sutured over the medial flap and into the breast tissue

**Fig. 11:**
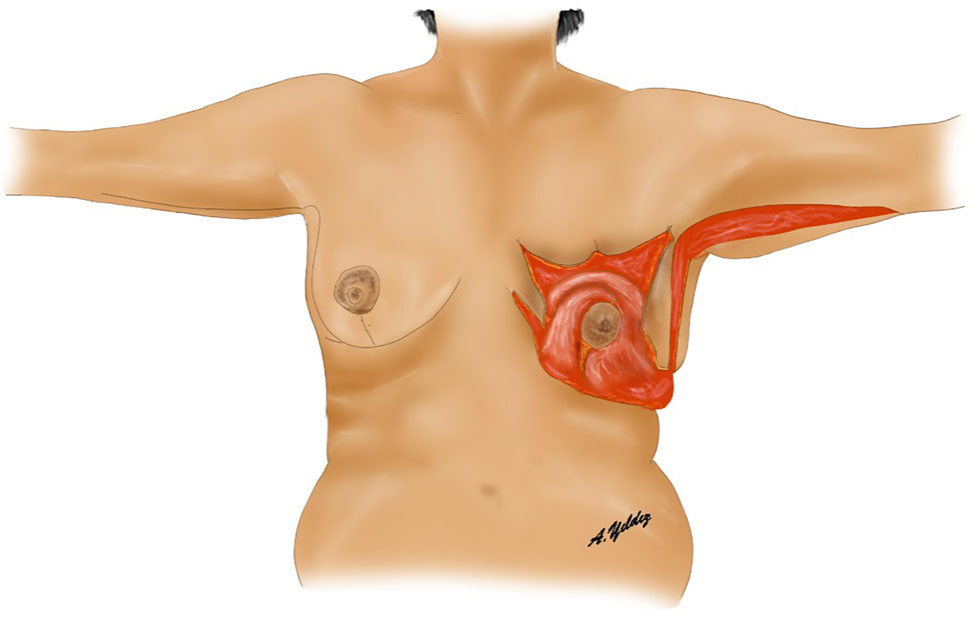
Left breast: lateral and medial flaps are adapted by folding over the central breast tissue. Right breast: illustration after skin closure

**Fig. 12:**
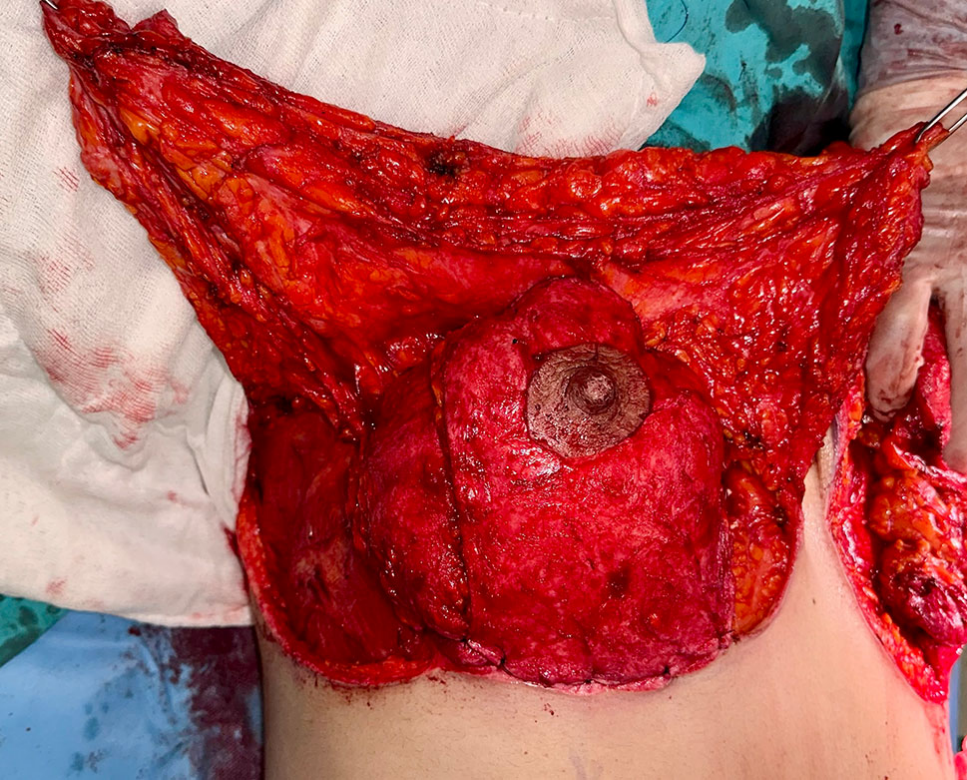
By parenchymal reshaping, the breast is given a round aesthetic appearance

**Fig. 13:**
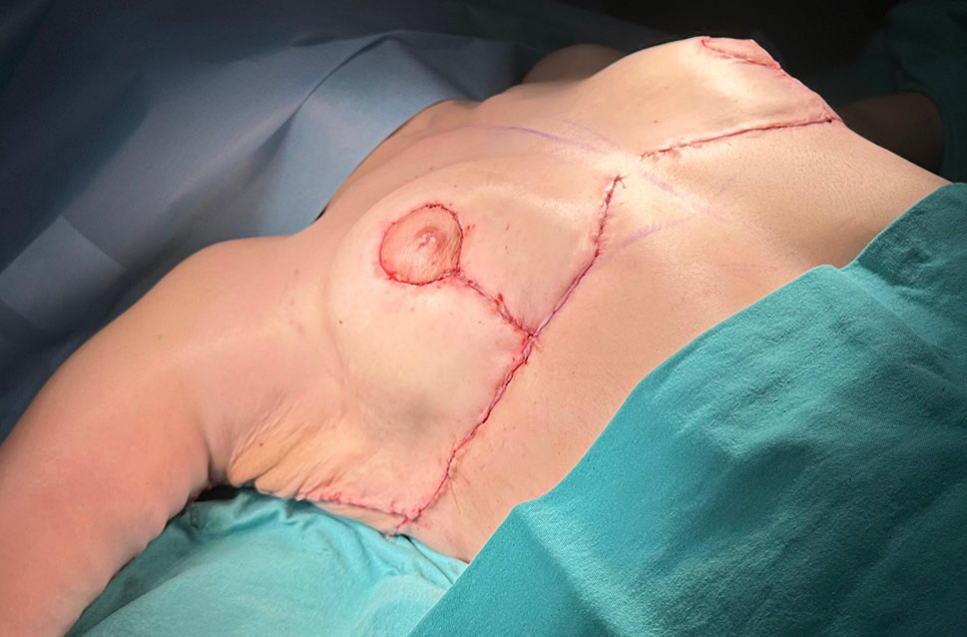
The skin is tailored and redraped over the mound

The patient is then positioned supine and all the breast quadrants excluding the NAC are deepithelialized (Fig. 9a). Thin subcutaneous mastectomy skin flaps are elevated (Figs. 5, 9b-left breast). Dissection is continued superiorly until the second rib. The inferior and central pedicles of the breast are preserved. Medially, the dermoglandular flap supplied by the anterior intercostal perforators is elevated over the pectoralis muscle layer (Figs. 1b,5). The central and inferior pedicled breast tissue is anchored at the level of the second rib perichondrium with a braided permanent suture. The lateral dermoglandular flap is supplied by the lateral thoracic and intercostal perforators (Fig. 1a). This lateral chest roll flap is folded over the central breast tissue and sutured over the medial flap (Figs. 10,11-right breast). The dermoglandular flaps are fixed to the third rib perichondrium. Parenchymal reshaping is initiated after dermal suspension is complete. This process involves shaping the parenchyma laterally, inferiorly and medially, forcing projection centrally with interrupted and running absorbable sutures (Fig. 12). The skin is draped over the reshaped breast tissue (Fig. 13). After resection of excess skin, the skin is closed in two layers with an absorbable suture (Fig. 11-left breast).

**Fig. 14:**
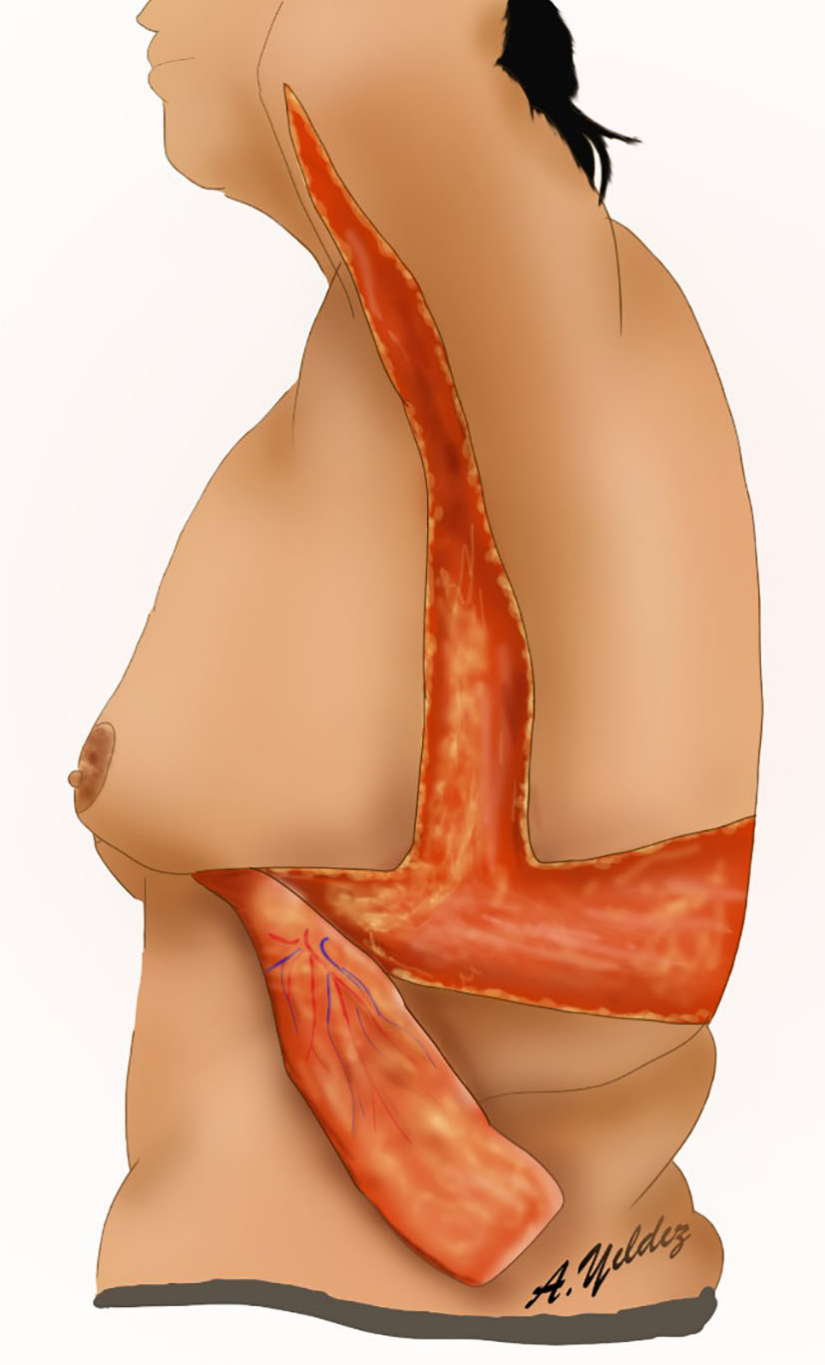
Lateral extended deepithelialized fasciocutaneous flap, lateral chest lift and arm lift line are illustrated

For the arm and lateral chest, the arms are abducted 90 degrees. After injecting tumescent to the arm and axillary region, liposuction is performed. Excess skin on the arm, axillary and lateral trunk is then resected over the superficial fascia (Fig. 14). The skin is closed in two layers with an absorbable suture.

## Results

A total of 24 patients were included in this study. The patients’ ages ranged from 23 to 45, with a mean age of 31.3 years (Table 1). Body mass index (BMI) ranged from 20.5 and 29.2 kg/m^2^, with a mean of 24.7 kg/m^2^. All patients had undergone bariatric surgery. The body contouring surgery was performed an average of 2.2 years after bariatric surgery. During this period, patients lost between 62 and 100 kg (mean 77.28 kg). Patients’ weights were stable for at least six months to be included in the study.

**Table 1:** Patient characteristics and outcome

Patient	Age, years	Operation interval^†^ (months)	Weight loss (kg) (before-after)	BMI (kg/m^2^)	Jugulum–nipple (cm)	Pittsburg scale	Operation	Complications	Follow-up (months)
1	26	60	75 (146-71)	25,5	R: 24, L: 24	Grade 3	AM+BB+LB	None	12
2	30	44	100 (155-55)	24,4	R: 27, L: 26	Grade 3	AM+BB+LB	None	7
3	45	43	79 (140-61)	25,7	R: 24, L: 24	Grade 3	AM+BB+LB	None	32
4	31	36	80 (145-65)	27,3	R: 31.0, L: 31.5	Grade 3	AM+BB+LB	Detachment at ‘T’	45
5	43	37	76 (140-64)	23,5	R: 29, L: 30	Grade 3	AM+BB+LB	None	19
6	23	30	89 (159-70)	27,9	R: 30.5, L: 29.5	Grade 3	AM+BB+LB	None	36
7	35	25	98 (150-52)	21,1	R: 26, L: 26.6	Grade 3	AM+BB+LB	None	32
8	27	33	77 (141-64)	27,2	R: 30.5, L: 29.5	Grade 3	AM+BB+LB	None	60
9	42	18	70 (128-58)	22,7	R: 31, L: 34.5	Grade 3	AM+LB	None	60
10	23	36	62 (145-83)	23,6	R: 27, L: 29	Grade 2	AM+LB	None	32
11	31	45	80 (130-50)	20,5	R: 26, L: 27	Grade 3	AM+LB	Detachment at ‘T’	12
12	25	38	93 (150-57)	25,2	R: 31.5, L: 31	Grade 2	AM+LB	None	1
13	38	29	95 (146-51)	21,7	R: 29.5, L: 30.5	Grade 3	AM+LB	None	37
14	40	34	64 (135-71)	28,9	R: 26, L: 27	Grade 3	AM+LB	None	22
15	36	24	73 (145-72)	27,6	R: 29.5, L: 28.5	Grade 2	AM+BB	None	21
16	29	35	70 (135-65)	27,7	R: 25.5, L: 25.5	Grade 3	AM+BB	None	20
17	27	31	63 (126-63)	25,9	R: 30.5, L: 31.5	Grade 3	AM+BB	None	33
18	30	27	82 (142-60)	21,4	R: 29, L: 30	Grade 2	AM+BB	None	20
19	31	24	87 (157-70)	26,3	R: 30, L: 29.5	Grade 2	AM+BB	None	21
20	33	42	84 (151-67)	24,7	R: 26, L: 26	Grade 3	AM+BB	None	31
21	29	37	64 (124-60)	23,5	R: 29, L: 29	Grade 2	AM	None	30
22	41	24	90 (168-78)	27,3	R: 36, L: 36	Grade 2	AM	None	60
23	24	14	63 (123-60)	22	R: 29, L: 31	Grade 3	AM	None	18
24	39	28	69 (142-73)	28,2	R: 27.5, L: 28	Grade 2	AM	Detachment at ‘T’	13

BMI, body mass index; AM, autoaugmentation mastopexy; BB, bra-line backlift; LB, l-brachioplasty; R, right; and L, left.

^†^Interval between bariatric operation and upper body lift

**Fig. 15:**
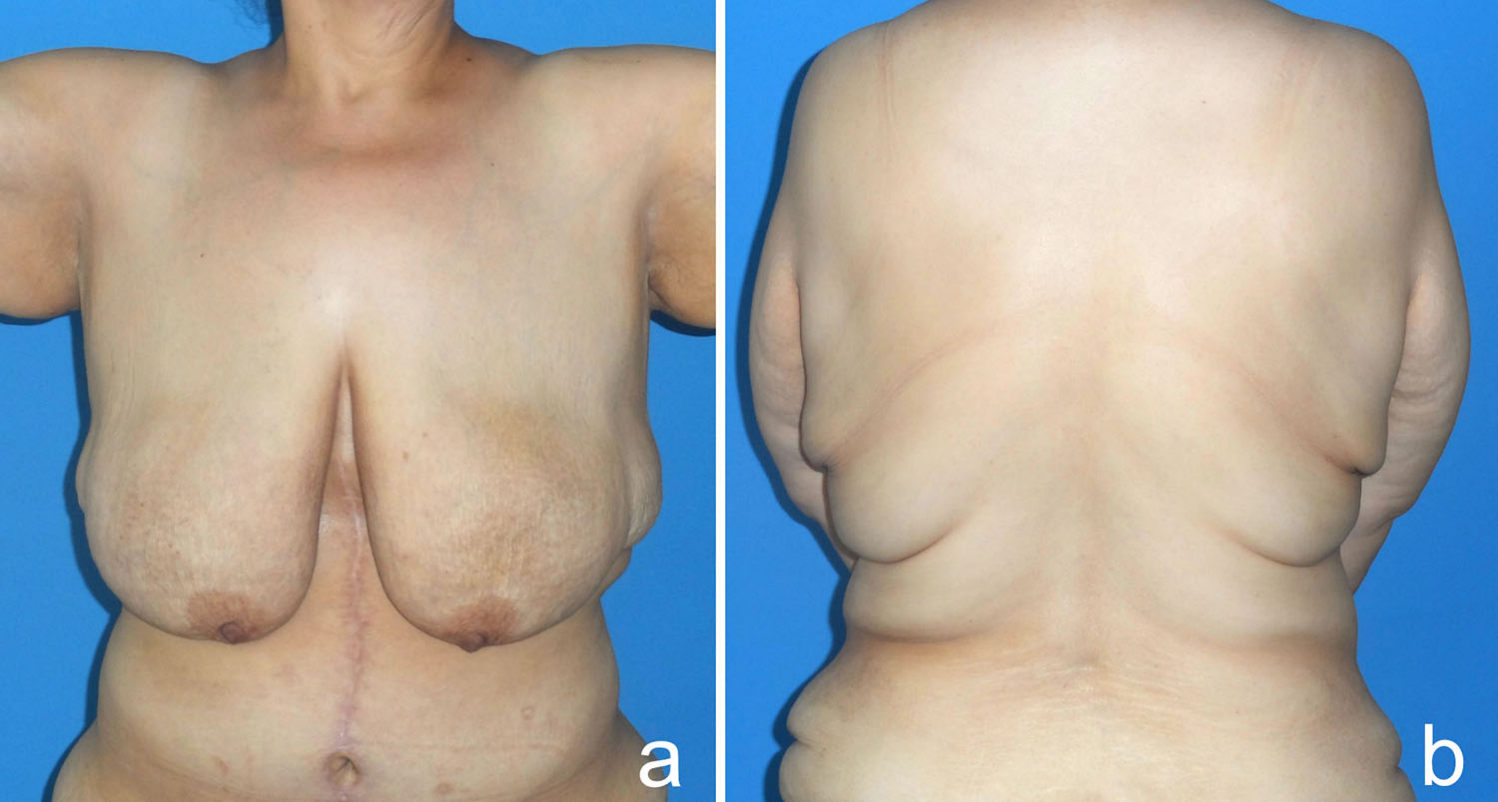
Patients typically present with pronounced breast ptosis, loss of projection in the upper pole and medialization of the NAC. In addition, most patients have a prominent lateral chest roll that blurs the border between the lateral breast and the chest wall. Folds are also present on the back

**Fig. 16:**
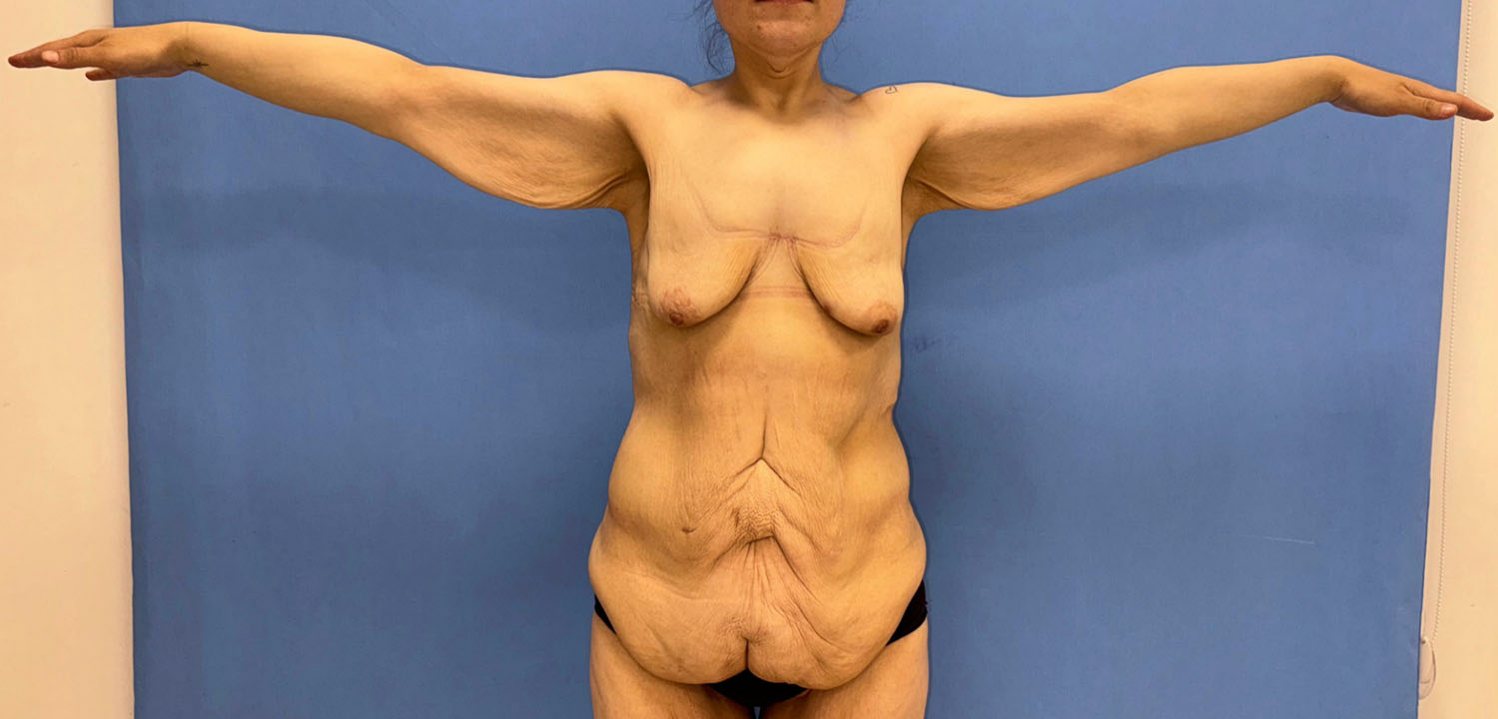
Patients have hanging skin in the arms and axillae

Upon physical examination, MWL patients exhibit decreased skin quality and diminished natural elasticity. They typically present with significant breast ptosis, loss of projection in the upper pole and medialization of the NAC. In addition, most patients have a noticeable skin fold in the lateral chest that distorts the border between the lateral breast and chest wall. Patients have multiple folds in their backs and sagging skin in their arms (Figs. 15, 16). The mean distance between the sternal notch and nipple was measured to be 28.8 cm (Table 1). According to the Pittsburgh scale (Table 2), 8 patients were evaluated as grade 2 and 16 patients as grade 3 (Table 1) [16].

**Table 2:** Pittsburgh rating scale

Grade	Scale
0	Normal
1	Ptosis grade I/II or severe macromastia
2	Ptosis grade III or moderate volume loss or constricted breast
3	Severe lateral roll and/or severe volume loss with loose skin

In this study, autoaugmentation with extended dermoglandular flaps procedure was performed in combination with other upper body lifting procedures according to the patient’s needs and preferences. In eight patients, ‘bra-line’ backlift and L-brachioplasty were combined with an autoaugmentation surgery. In six patients, the autoaugmentation procedure was combined with L-brachioplasty. In six patients, the autoaugmentation procedure was combined with ‘bra-line’ backlift. In four patients, autoaugmentation was the only performed procedure (Table 1).

Patients were mobilized in the early postoperative period. Drains were removed on the third postoperative day on average. Patients were followed up between one month and five years. In the early period, detachment occurred in the ‘T’ junction in three patients. For these patients, no surgical intervention was required as the detachment sides were treated with dressing and full recovery was succeeded. No nipple necrosis, skin flap necrosis, fat necrosis, seroma or hematoma were observed in any patient. Breast ptosis recurrence occurred in 18% of patients in the long term. However, these patients were satisfied with their results and did not request for a revision.

**Fig. 17:**
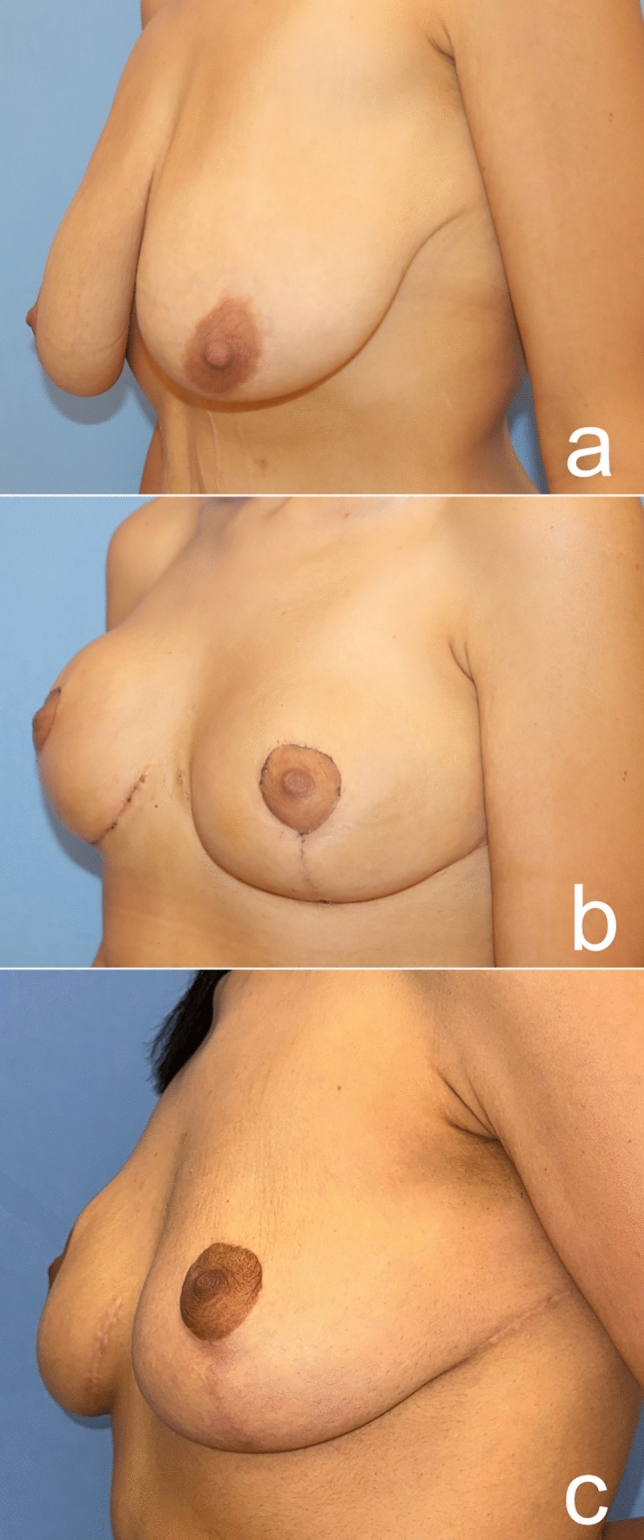
A 24-year-old patient was autoaugmented with a dermoglandular flap, 1.5 years after the bariatric procedure. Preoperative (**a**), postoperative first month (**b**), postoperative sixth month (**c**) images of the patient

**Fig. 18:**
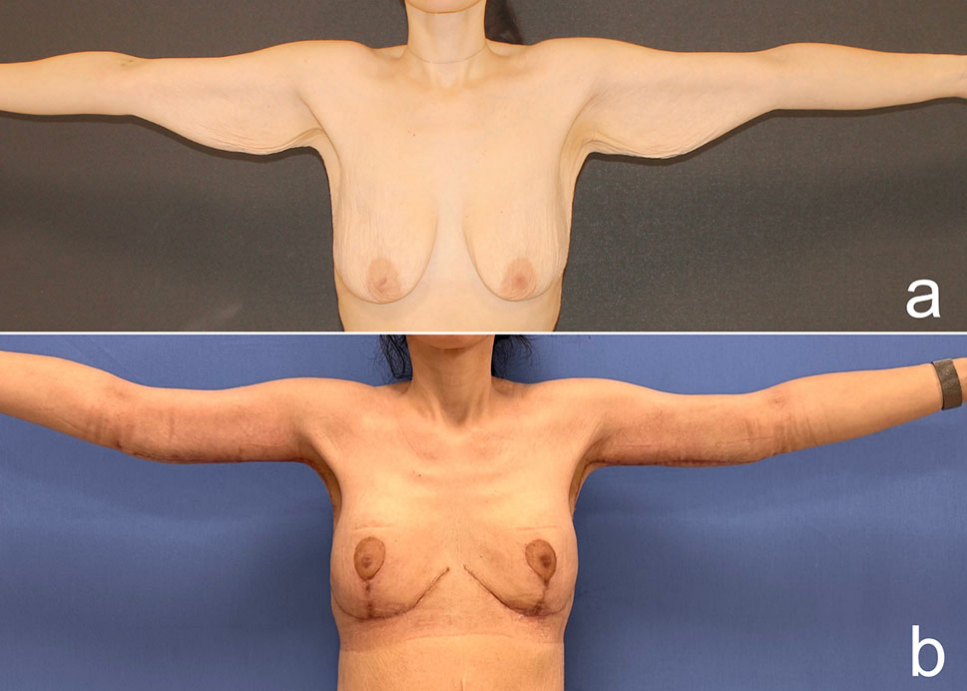
Pre- (**a**) and postoperative third-month images (**b**) of a 30-year-old patient who lost 80 kg after a bariatric procedure and underwent autoaugmentation and brachioplasty

**Fig. 19:**
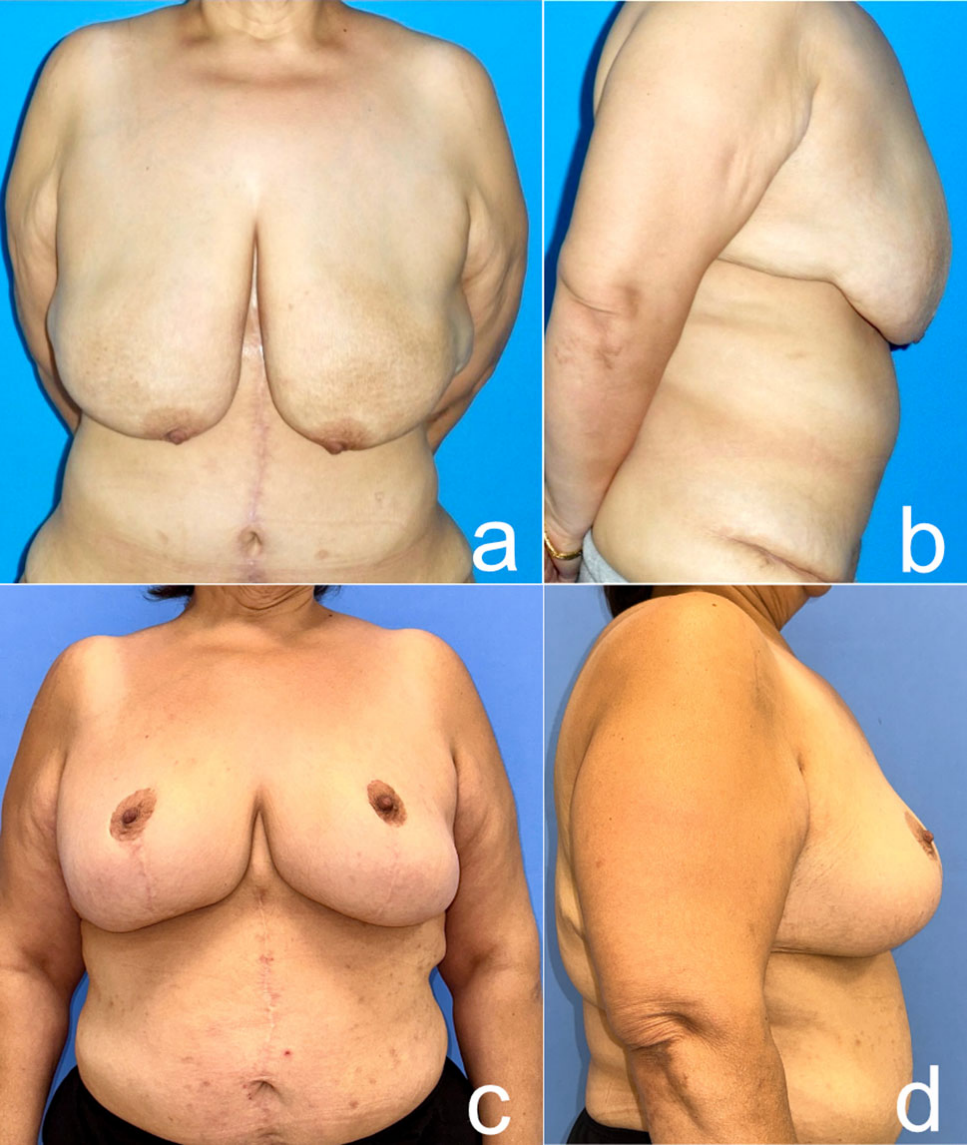
Before (**a** frontal view, **b** side view) and five years after (**c** frontal view, **d** side view) autoaugmentation and brachioplasty of a 41-year-old woman who lost 90 kg after a bariatric procedure. The nipple is still as high as it should be after a long time, but the patient has gained weight and this has affected the final result

**Fig. 20:**
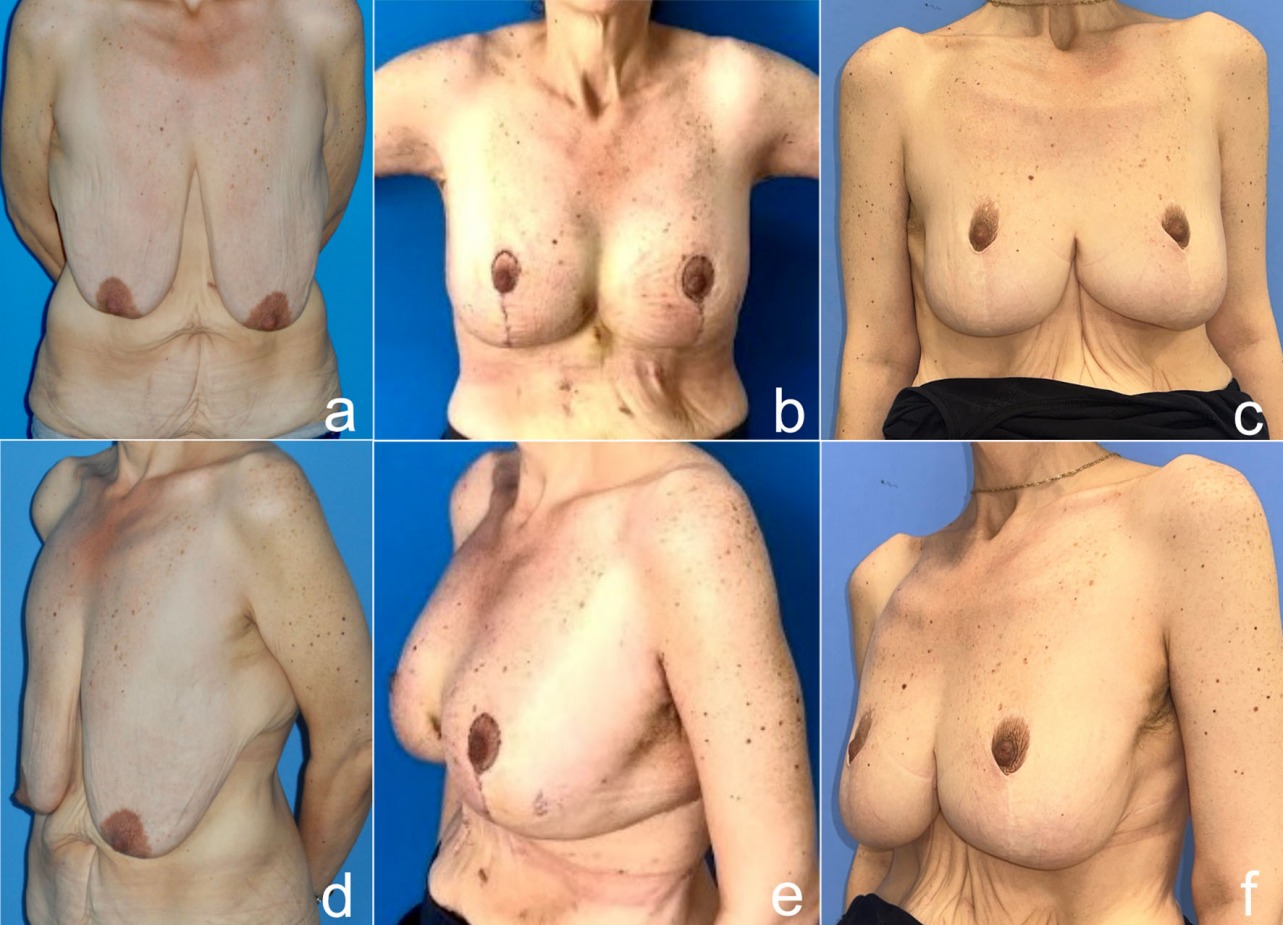
Before (**a** frontal view, **d** oblique view), one month after (**b** frontal view, **e** oblique view) and five years after (**c** frontal view, **f** oblique view) autoaugmentation and brachioplasty of a 43-year-old woman who lost 70 kg after a bariatric procedure. The nipple is where it should be, but pseudoptosis has developed due to weight loss and time. However, the patient did not accept the revision surgery, because she was satisfied with the result

As a result of this procedure, autoaugmentation of the breast was achieved by using the lateral chest- and dorsal fold. The desired breast projection was achieved with the autologous tissue. The gap in the upper pole was filled permanently. Mastopexy was sufficiently performed. The sagging of the back, lateral chest, axilla and arms was eliminated (Videos 1, 2, Figs 17, 18, 19, 20).

Reduction/Mastopexy Module of Breast-Q is a powerful tool to measure quality of life in post-surgery phase. In the study, postoperative scales of Reduction/Mastopexy Module have been used in 23 patients that have been followed up at least 6 postoperative months to analyze satisfaction and quality of life with respect to psychological well-being, sexual well-being, physical well-being, satisfaction with breasts, satisfaction with outcome, satisfaction with information, satisfaction with surgeon and satisfaction with medical team. As results shown in Table 3, an overall well-being score of 87 and overall satisfaction score of 96 have been accomplished.

**Table 3:**
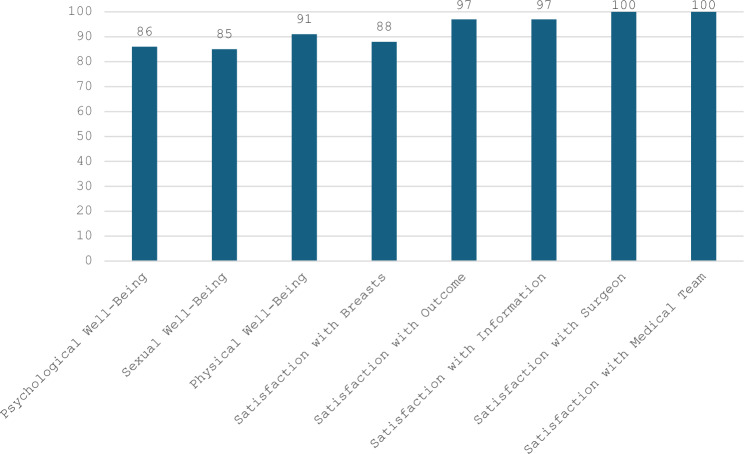
Reduction/mastopexy module of BREAST-Q scores

## Discussion

In the past decade, bariatric procedures have gained popularity as a surgical treatment for obesity [17]. While bariatric surgery can manage excess weight and many of its comorbidities, excess skin and body deformities remain unaddressed. After MWL, an increasing number of women have started to demand breast reshaping procedures. MWL patients usually have significant volume loss at the breast, and the ligamentous support of the breast is significantly compromised. This usually leads to a ptotic, deflated breast with severe upper pole deficit, medialized NAC and a lowered inframammary fold (IMF) position. Furthermore, the lateral curvature of the breast is frequently lost because of the redundant lateral chest tissue (Fig. 15). Last but not least, skin sagging in the whole upper body, including the arm, axilla, lateral thoracic and back, is observed due to inflexible and lax skin (Fig. 16).

Following bariatric weight loss, contour deformities have been categorized using the Pittsburgh rating scale [16]. Depending on the severity of the deformity, the breasts have been assigned to four simplified grades ranging from 0 to 4, as shown in Table 1. In the study, 8 patients (33%) were classified as grade 2 and 16 patients (77%) as grade 3.

**Fig. 21:**
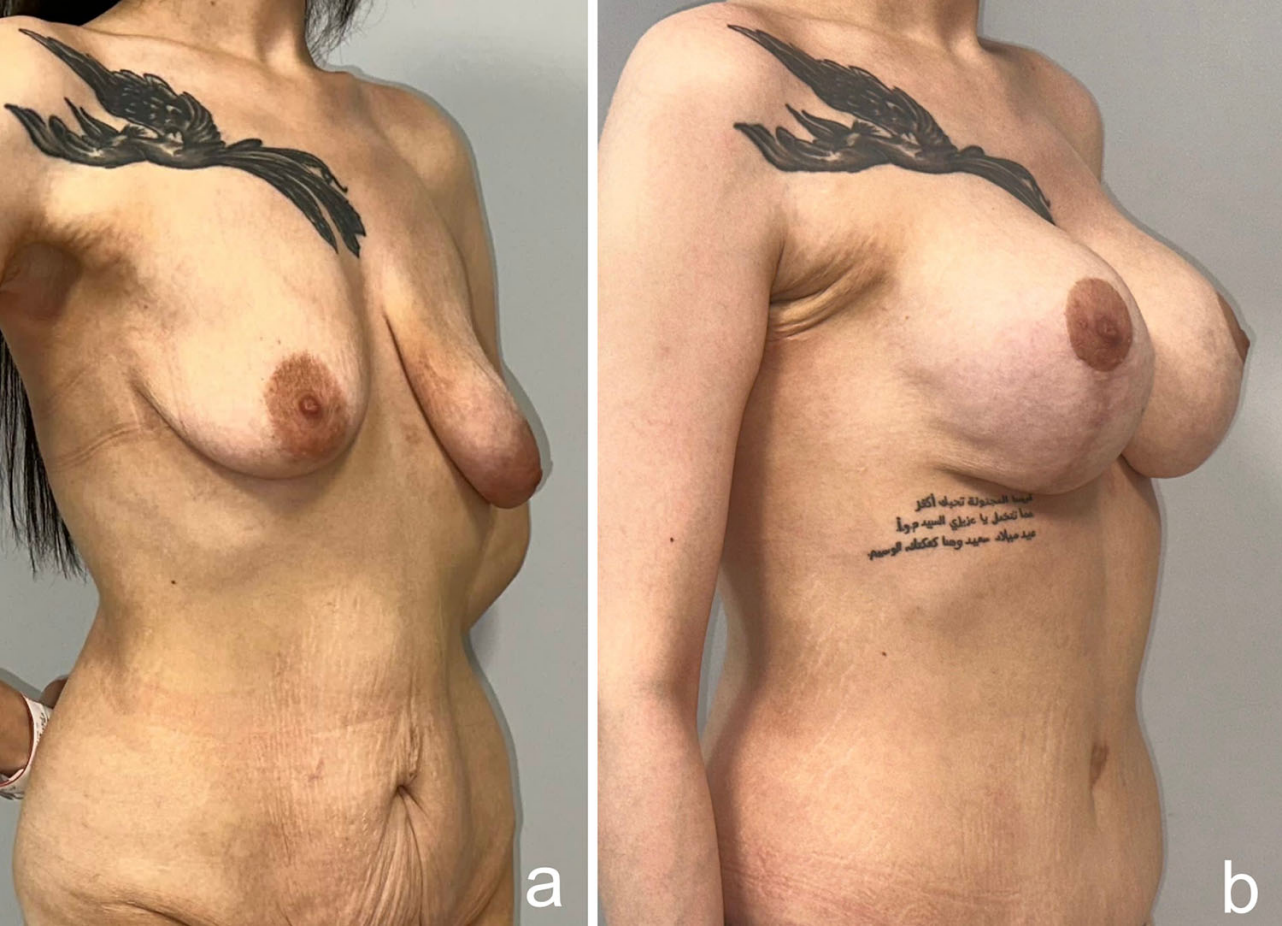
**a** A 27-year-old female patient lost 65 kg after bariatric procedure. **b** 1 year after augmentation mastopexy with implant operation

The goals of mastopexy following MWL include achieving a well-contoured breast with upper pole fullness, an appropriate nipple position, and a result that sustains its shape over time. While correcting the deflated breasts, other aesthetic units of the upper body should also be evaluated. In order to achieve a satisfactory result not in multiple stages but just in a single stage, the plastic surgeon can perform mastopexy combining lateral chest-, arm- and backlift procedures. The treatment should be tailored based on patient’s desires, breast shape, lateral chest rolls, arms and back. MWL patients should be thoroughly counseled regarding revision surgeries for recurrent ptosis due to skin quality and laxity. Breast deformity in these patients can be treated by traditional mastopexy alone or mastopexy with implants. Implants are often an appealing option for patients and surgeons. When compared to autologous procedures, these require less operating time, have a faster postoperative recovery and avoid donor-site morbidity. While autologous methods have a limit to the volume that can be achieved, implants can offer higher volumes. However, these sagging breasts adapt poorly to implants and leads rarely to complications such as implant exposure, implant malposition and uncertainty of the areola’s final position due to skin laxity [18]. Although implant placement in MWL patients is technically challenging, satisfying results can of course be achieved (Fig. 21). Autologous reconstruction may prevent implant-related complications such as capsular contracture, rippling, implant migration and breast implant illness. Furthermore, the outcomes of autologous procedures may age with the patient in a more natural way. For patients who do not want implants for various reasons, we can use autologous methods from our toolbox.

**Fig. 22:**
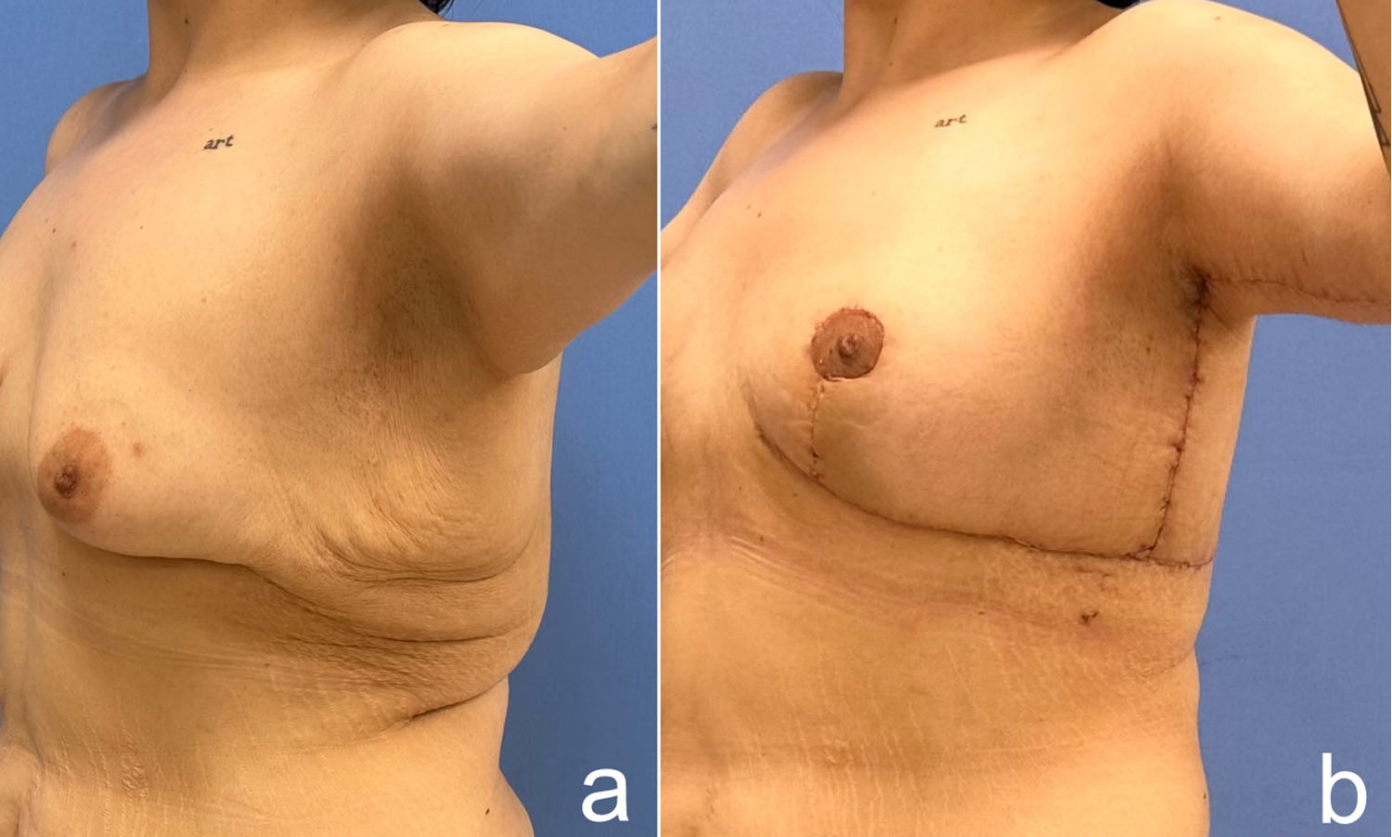
In this technique, lateral chest rolls are resected in every patient whether or not a concomitant procedure is performed

The presented autologous augmentation technique utilizes excess tissue from the lateral chest and/or back. As shown in Fig. 6, the red hatched flap on the lateral chest back can be extended safely further to the back midline, as shown by the red arrows, according to the patient. Or the flap on the lateral chest can be extended superiorly, as shown by the blue arrows (Fig. 6). This serves two purposes. Firstly, it removes the lateral chest- and back rolls and serves as a back-lift technique (Fig. 22). Secondly, these tissues are used to augment the deflated breast. This strategy is also applied to gluteal augmentation in the MWL patients [19, 20]. Before adapting the flap to breast tissue, perfusion is checked by trimming the distal tip. The rich perforator network in the lateral thoracic region and the large caliber pedicles in MWL patients make it possible to harvest flaps safely. Therefore, flap necrosis or fat necrosis were not occurred in this study. The reliability of the distal flap was verified with indocyanine green angiography (Fig. 8).

This procedure can be combined with an upper body lift by extending the lateral incision on to the back or with a brachioplasty by curving the incision up toward the axilla. In this study, 79% of patients underwent concomitant procedures. No significant early or late complications occurred. According to a cohort study by Rubin, operations were performed concomitantly with a mastopexy (93.4%). In particular, mastopexy has been combined with abdominoplasty (79%) [21]. According to a study by Losken and Holtz, 35 MWL patients underwent a breast contouring procedure. 80% of their patients underwent concomitant procedures performed [22]. This suggests that mastopexy is a safe operation when combined with other procedures.

For longer-lasting results, dermal fixation of the central breast tissue to the perichondrium of the second rib rather than the pectoralis fascia preserves the upper pole fullness for longer [12]. Due to the fact that the lower pole is not emptied and the central and inferior pedicles are used, the breast tissue may bottom out over time and pseudoptosis may occur. Therefore, planning the NAC height lower than the ideal breast measurements (NAC to sternal notch 18–21 cm) can prevent the breast tissue from bottoming out. Thus, too much volume will be suspended less. Biologic mesh materials can be used to enhance durability, however, at an additional financial cost.

If we consider UBL surgery as the first stage of metamorphosis in MWL patients, the other stages need to be planned at least three months later. These stages include lower body lift, gluteal contouring, thigh lift, mons reduction or liposuction, depending on the patient. In this journey, if a bottoming out is seen, the inferior breast tissue can be tucked up at every stage.

Treating the arm, axillary region, lateral thorax, back and breasts as a single unit creates harmonious results in the upper body. Despite the considerable operating time, overall these combined procedures reduce the number of operations and produce sustainable results in the long term. This means lower financial cost, less hospitalization time, a reduced overall anesthesia rehabilitation and downtime for recovery. We discovered that these patients have a better chance of achieving their desired body sooner. However, this procedure has some drawbacks. The more extensive tissue manipulation increases the risk of postoperative wound healing problems. With the patient positioning from supine to prone intraoperatively and extensive deepithelialization of a wide area, the operation takes a considerable amount of time. However, the operation time can be shortened by mechanizing deepithelialization with instruments such as the Padgett dermatome. For thromboembolic prophylaxis, graduated compression stockings and intermittent pneumatic compression devices need to be used. After bariatric procedures, patients tend to have deficiencies that delay wound healing.

## Conclusion

In MWL patients, focusing only on the augmentation mastopexy of the breasts leads to aesthetic disharmony in the upper body. The strategy of using the reserved subcutaneous tissue in autologous augmentation provides both upper body lift in a single session and gives the breast a more desired volume. The patients’ five-year follow-up results show that this technique offers long-lasting results with low complication and recurrence rates. This approach is autologous, cost-effective, feasible and reliable, as it minimizes the number of operations.

## Supplementary Information

Below is the link to the electronic supplementary material.

Supplementary file 1 Video 1: 360-degree video of a 26-year-old female patient before (top) and three months after (bottom) upper body lift surgery, following a weight loss of 75 kg through a bariatric procedure (MP4 19119 KB)

Supplementary file 2 Video 2: 360-degree video of a 30-year-old female patient before (top) and three months after (bottom) upper body lift surgery, following a weight loss of 100 kg through a bariatric procedure (MP4 7195 KB)
